# Detection of pathogenic leptospires in water bodies in neighborhoods vulnerable to flooding, through a participatory research approach in Santa Fe (Argentina)

**DOI:** 10.1371/journal.pgph.0006447

**Published:** 2026-06-08

**Authors:** Julieta V. Carletti, Eva C. Rueda, Juliana Sesma, Maximiliano A. Cristaldi, Tamara Ricardo, Celeste Medrano, Christian A. Avalos, Diego A. Mendicino, Maria Andrea Previtali

**Affiliations:** 1 Consejo Nacional de Investigaciones Científicas y Técnicas (CONICET), Santa Fe, Argentina; 2 Dpto. de Ciencias Naturales, Facultad de Humanidades y Ciencias (FHUC), Universidad Nacional del Litoral (UNL), Santa Fe, Argentina; 3 CAECIHS, Universidad Abierta Interamericana, Rosario, Argentina; 4 CONICET, Rosario, Argentina; 5 Facultad de Ciencias Médicas, Universidad Nacional de Rosario, Rosario, Argentina; 6 Hospital Provincial de Rosario, Rosario, Argentina; 7 Instituto de Ciencias Antropológicas, Universidad de Buenos Aires, Buenos Aires, Argentina; 8 Centro de Investigaciones sobre Endemias Nacionales (CIEN), Facultad de Bioquímica y Ciencias Biológicas (FBCB), Universidad Nacional del Litoral (UNL), Santa Fe, Argentina; New York University Grossman School of Medicine, UNITED STATES OF AMERICA

## Abstract

This study aimed to identify areas with the presence of pathogenic *Leptospira* in marginalized neighborhoods of Santa Fe, Argentina, through a participatory research approach. Additionally, we aimed to optimize a low-cost and sensitive detection method suitable for public sector laboratories in resource-limited settings. Three neighborhoods highly vulnerable to hydrological risks and leptospirosis outbreaks—Colastiné Sur, La Vuelta del Paraguayo, and Chalet—were selected. A series of meetings with local residents were conducted to discuss the risks of leptospirosis and to collaboratively map high-priority water sampling sites based on local knowledge and perceived exposure. Community members then participated actively in sample collection and documentation of environmental conditions. In parallel, we optimized a DNA extraction and qPCR protocol tailored for complex environmental matrices, enabling the sensitive detection of pathogenic *Leptospira*. *Leptospira* DNA was detected in 21.1% of the 33 water samples collected: 9.09% in La Vuelta del Paraguayo, 18.18% in Chalet, and 36.36% in Colastiné Sur. The results were subsequently shared and discussed with community members, fostering a dialogue between scientific findings and local knowledge. This study is the first of its kind in Santa Fe and highlights the dual value of community engagement in environmental health surveillance and the implementation of an affordable and robust molecular tool for leptospirosis monitoring. Our findings illustrate how integrating participatory mapping with environmental sampling can guide the molecular detection of *Leptospira* in urban water bodies—an approach that has rarely been documented—and underscore the potential of combining local knowledge and accessible molecular tools to strengthen public health responses in flood-prone settings.

## Introduction

In public health research, the groups under study are typically seen as passive subjects who are studied and reported on without active involvement in the research process. This traditional approach has been questioned both for its inability to suppress researcher’s bias and for how it objectifies and disempowers communities [1]. Participatory approaches have increasingly been incorporated into epidemiological and public health research as a way to integrate scientific knowledge with the experience and contextual understanding of local communities. In participatory epidemiology, community members and other stakeholders contribute to identifying health problems, interpreting local environmental conditions, and generating knowledge that can inform disease surveillance and prevention strategies [2]. Such collaborations can improve the identification of environmental and social determinants of disease and facilitate the translation of research findings into locally meaningful prevention strategies [3]. These participatory approaches foster collaborative partnerships among researchers, public health institutions, and community organizations, allowing the co-production of knowledge that is both scientifically robust, locally relevant, transferable and applicable elsewhere [4,5]. The co-production of knowledge has been shown to improve the quality and acceptability of health policies across different sectors, being particularly effective in promoting adherence to zoonotic disease prevention measures [6]. It allows for the identification of biases in access to health information, and contextualizes that information for the community, enhancing the understanding of social and environmental determinants of health [6]. The participation of residents can also contribute to a more contextualized socio-ecological understanding of disease risk by incorporating local knowledge about environmental conditions, daily practices, and places where exposure is more likely to occur [7].

Leptospirosis, a zoonotic disease caused by bacteria of the genus *Leptospira*, affects over one million people and causes more than 60,000 deaths annually [8]. Pathogenic leptospires are shed in the urine of infected mammals and can survive in water or moist soil for weeks, especially in warm environments [9]. Humans are typically infected by contact with water or soil contaminated with the bacteria [9]. Leptospires are mobilized during heavy rains or floods, often triggering leptospirosis outbreaks due to increased exposure to water and mud [10,11]. Warm temperate areas with abundant water bodies are particularly at risk [12]. Identifying habitats that can serve as reservoirs for the bacteria is fundamental to define the environments that pose the greatest risk to the population and to target public health policies in this direction.

Although leptospirosis occurs in a wide range of social conditions in both urban and rural areas, it mainly affects marginalized or vulnerable populations, particularly those living in precarious housing, or residing in proximity to micro-dumpsites or flood-prone areas [10,13,14]. In Argentina leptospirosis represents a major public health issue, and several outbreaks and epidemics have occurred, mainly affecting people from rural areas or after floods [15]. The province of Santa Fe is among the Argentine jurisdictions with the highest number of confirmed and probable leptospirosis cases recorded each year. In 2025, 46 cases were reported in the province, accounting for 34% of the country's total [16]. Outbreaks of leptospirosis are recurrent in Santa Fe, especially during periods of heavy rainfall and flooding [17]. In Santa Fe, *Leptospira* infection was also detected in domestic and synanthropic animals [18,19].

In the present study, we aimed to create a collaborative network to identify areas with a high likelihood of the presence of *Leptospira*. The initiative involved neighbors, members of local civil associations (Revuelta, La Garganta Poderosa, and Biblioteca de Las Orillas), professionals from different disciplines and institutions, including researchers from the Universidad Nacional del Litoral and public health personnel from the Ministry of Health. This research also aims to optimize protocols for detecting pathogenic leptospires in environmental water samples, which are complex due to the presence of various inhibitors and contaminants that can reduce PCR efficiency. Our goal was to integrate community participation and scientific expertise to address the environmental and social dimensions of leptospirosis in sectors of the city of Santa Fe with high suitability for the occurrence of leptospirosis [20]. In this context, participatory processes can help identify locally relevant environmental sites and practices associated with exposure, thereby guiding targeted environmental surveillance using molecular tools for pathogen detection.

## Methodology

### Study setting

The city of Santa Fe is located at the confluence of two major river systems: the Salado River to the West and the Paraná River to the East. This geographic location makes Santa Fe highly vulnerable to hydrological hazards, including river flooding, heavy rainfall, or their combination. Leptospirosis outbreaks have been linked to the Salado River flood in 2003, the Paraná River floods in 2010 and 2016, and an extreme rainfall event in 2007 [15,21,22].

**Fig 1:**
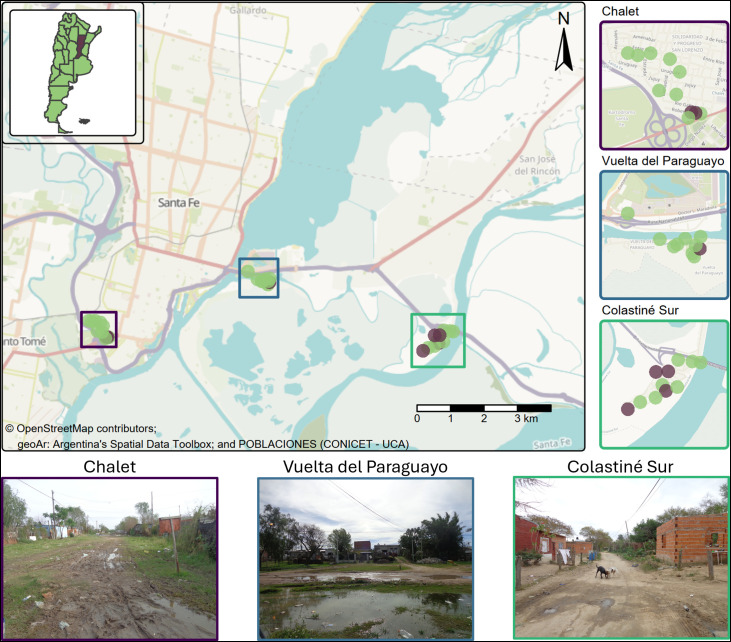
Location and views of the study sites in Santa Fe city, Argentina. The main panel shows the spatial distribution of the study sites within the city, while the inset map indicates the location of Santa Fe Province within Argentina. Panels on the right provide enlarged views of each study site. Purple circles represent positive samples and green circles represent negative samples. Base maps were obtained from OpenStreetMap contributors, and vector layers were generated using layers from poblaciones.org and the geoAR R package. Panels on the bottom are pictures taken by MAP showing some of the deficiencies in infrastructure and services that increase the risk for the occurrence of leptospirosis in the neighborhoods that participated in the study: (CH) Chalet, (LVP) La Vuelta del Paraguayo and, (CS) Colastiné Sur.

The city of Santa Fe includes 69 popular neighborhoods (Ministerio de Desarrollo Social [23]), primarily concentrated in the western and riverside areas [24]. Three of these neighborhoods were selected for this study: Chalet (CH), La Vuelta del Paraguayo (LVP), and Colastiné Sur (CS) (Fig 1). These neighborhoods face deficiencies in infrastructure and services that increase the risk for the occurrence of leptospirosis, including unpaved roads, irregular garbage collection, lack of sewage systems, open drains and channels, and, in the case of CS, lack of access to piped water (Fig 1). All three areas of the city are flood-prone, either due to heavy rainfall or, in the case of LVP and CS, to overflows of lagoons or of effluents of the Paraná River, the Riacho Santa Fe and the Colastiné river, respectively.

CH is located in the southwestern part of the city (Fig 1) and has 3,000 inhabitants. Only a quarter of the population has formal employment, while the rest engage in informal jobs, such as cardboard collection and rag-picking [25]. LVP is a periurban neighborhood of approximately 130 families (equivalent to about 500 inhabitants) located on an island to the east (Fig 1). Many residents rely on subsistence fishing, hunting, and straw collection as their primary activities or as supplements to other jobs. The proximity to the river, combined with inadequate infrastructure, leads to occasional flooding. In addition, the lack of road grading and proper drainage makes the main dirt road impassable even during regular rainfall.

CS is located to the east by the Colastiné River (Fig 1), and it is home to approximately 700 inhabitants, some of whom depend on subsistence fishing and hunting. The main concerns of the neighbors are related to the lack of access to piped water and the need for maintenance of the levee built to prevent flooding caused by river overflow.

### Study design

**Fig 2:**
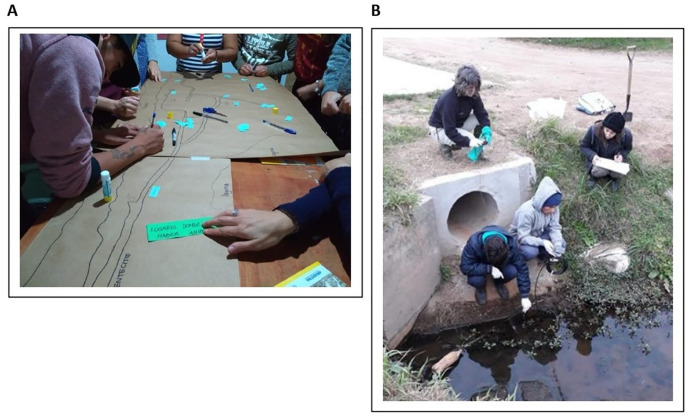
Involvement of the community in different moments of the study, A) during collective mapping for the identification of sites of interest as potential risk of leptospirosis, and B) during the sampling efforts on the hotspots identified on the collective mapping exercise. The individuals pictured in these images have provided written informed consent (as outlined in PLOS consent form) to publish their image alongside the manuscript.

Between May and June 2019, we conducted a participatory research and cross-sectional study that involved five workshops that moved from initial discussions of neighborhood history, environmental conditions, and health concerns, to collective identification of potential leptospirosis risk locations, planning of environmental sampling, and the subsequent sharing and discussion of laboratory findings with participants. Participation in these workshops varied depending on the activity; approximately 8–12 participants (neighbors and local health personnel) were involved in each neighborhood, with most attending collective mapping sessions and smaller groups participating in field sampling activities. These workshops took place at the Bachillerato Popular, a folk high school for adults in LVP, in CS they took place in a park, at a neighbor's house, and at the Public Library, while in CH they were held at the Health Center. These encounters began with a round of introductions, followed by the presentation of the research goal. We then discussed the main aspects of leptospirosis through the exploration of flyers, brochures and other resources. Later on, the mapping activity took place following the approach proposed by Risler & Ares [26]. A hand-drawn map of the neighborhood was placed on the table allowing the participants to freely explore the material and mark points of interest, such as schools, parks, churches, lagoons, rivers, etc. To stimulate discussion about environmental conditions associated with leptospirosis risk, a set of trigger questions guided the participatory mapping exercise. Participants were asked to use different icons to mark locations where rodents are frequently seen, areas with abundant stray dogs, places where livestock (pigs, horses or cows) are held, areas where large puddles or ponds are formed after the rain, location of garbage dumps, and places where people have contact with environmental water for recreational or work-related purposes (Fig 2A). The overlapping of several icons on the map helped to identify potential hotspots. Based on these collectively identified locations, environmental sampling sites were selected through discussion and consensus with participants, who were also invited to take part in the sampling activities. Participation in field sampling was also opened to other neighbors who were invited via a flier that circulated within neighborhood social media networks.

Collection of water samples took place in June 2019. During the sampling activities, participants spontaneously assumed different roles according to their interests and availability, including measuring pH and temperature, taking photographs in multiple directions, recording field data, and labeling sample tubes (Fig 2B). At each selected location, a sample of 100 ml of water was collected using two sterilized 50 ml Falcon tubes that were stored and transported in a refrigerated cooler. Sampling points were georeferenced using GPS, and environmental parameters such as water temperature, pH, dissolved oxygen, conductivity, salinity, and turbidity were measured using a multiparameter (YSI - model 85). For each sample, we collected environmental data, including the type of water source (river, lagoon or wetland, pond, roadside channel or ditch, flooded area, drainage, or other), the amount of light exposure (full light, semi-shade, or shade), and the presence of the following within 50–150 meters: stray dogs, stray/feral cats, poultry, cattle, horses, goats, or rabbits (yes/no for each). We also noted the presence of garbage within the same radius (yes/no), estimated its quantity (low, high), and documented the presence of debris (yes/no) and weeds (yes/no).

### DNA extraction

Immediately upon arrival at the laboratory, the two 50-ml water samples collected in each sample point were concentrated by filtration using a single 0.22-µm Millipore nitrocellulose MF membrane (Merck KGaA, Darmstadt, Germany). The membranes were stored at -20 °C until DNA extraction.

Genomic DNA extraction from the membranes was performed following the protocol of Vital-Brazil et al. [27]. Environmental DNA extracts were run on a 0.8% agarose gel stained with GelGreen in 0.5X TBE buffer at 120V for 30 minutes and visualized using a transilluminator to assess the quality of the extracted DNA based on the degradation level.

### PCR reactions for detection of *Leptospira*

To enhance sensibility of detection, we performed a Taqman assay targeting the LipL32 gene to detect the presence of pathogenic *Leptospira*, following the protocol by Schneider et al. [28] with minor modifications. Each reaction contained 10 μL of qPCR MasterMix (iTaq Universal Probes Supermix (Bio-Rad, Cat. No. 1725130), 500 nM of primers LipL32-45F and LipL32-286R, 100 nM of probe LipL32-189P (Table 1) and 3 μL DNA in a final volume of 25 μL. Amplification was performed using a real-time thermocycler (Bio-Rad CFX96) with the following program: an initial step of 2 minutes at 50˚C, followed by 2 minutes at 95˚C, and 44 amplification cycles (15 seconds at 95˚C and 1 minute at 60˚C). All samples were tested in duplicate; negative controls were included in each row of plates to detect any contaminating DNA. As a positive control, 150 ng of *Leptospira interrogans* serovar Canicola strain Hond Utrecht IV DNA (10^8^ bacteria/ml) was used, provided by the Leptospirosis Laboratory of the National Institute for Respiratory Diseases “Dr. E. Coni.” Samples with Cq values below 40 were considered positive.

**Table 1:** Primers and probes used for molecular detection of pathogenic leptospires in environmental water samples.

Primer/Probe	Sequence
*Real-time quantitative PCR*	
*LipL*32-45_F	5′ AAGCATTACCGCTTGTGGTG 3′
*LipL32*-286_R	5′ GAACTCCCATTTCAGCGATT 3′
*LipL32*-189_P	FAM-AAAGCCAGGACAAGCGCCG-BHQ1
*Nested PCR*	
SeqY II_F	5′ GAATTTCTCTTTTGATCTTCG 3′
SeqY IV_R	5′ GAGTTAGAGCTCAAATCTAAG 3′
G1_F	5′ CTGAATCGCTGTATAAAAGT 3′
G2_R	5′ GGAAAACAAATGGTCGGAAG 3′

Nested PCR was performed in qPCR positive samples to distinguish pathogenic species of *Leptospira* by targeting the SecY gene. The first reaction used SecYII and SecYIV primers (Table 1), followed by an inner reaction with primers G1 and G2 (Table 1), according to the protocol proposed by Casanovas-Massana et al. [29] with minor modifications. Each reaction contained 12.5 µL of Mint Master Mix 2X (Inbio HighWay), 400 nM of either the first or second primer pair (Table 1), and 3 µL of DNA extract or product in a total volume of 25 µL. Amplifications were performed in a thermocycler (Bio-Rad TX100 Touch Thermal Cycler) using the following program: 94 °C for 5 minutes, followed by 35 cycles of 94 °C for 30 seconds, 55 °C for 45 seconds and 72 °C for 60 seconds, with a final extension at 72 °C for 7 minutes.

### Sequencing

The qPCR products were run on a 2% agarose gel and purified using the QIAquick Gel Extraction Kit (QIAgen Cat no./ID 28704) following the manufacturer's instructions. The purified products were sequenced by the Sanger method using primers G1_F and G2_R. Sequences were edited using BioEdit 7.2.5 (Ibis Biosciences) and Macrogen Inc. service. The sequences obtained were compared with available ones in the GenBank database using the BLAST algorithm.

### Statistical analysis

Physico-chemical indicators were assessed for normality using the Lilliefors (Kolmogorov-Smirnov) test implemented in the Nortest package [30] and were described using either the mean and its 95% confidence interval (95% CI) or the median and interquartile range (IQR), as appropriate. The frequencies of samples positive to pathogenic *Leptospira* by study sites and environmental characteristics were compared using Pearson's Chi-squares test or Fisher's exact test for categorical variables and Kruskal-Wallis test of Wilcoxon rank test for numerical variables, using the package gtsummary [31] in the *R* software version 4.4.2 [19]. Temperature, dissolved oxygen and conductivity were categorized based on their quantiles. In addition, pH was categorized into three categories (acid, basic or neutral) depending on whether its value was below, higher or equal to 7, respectively. To deal with infrequent factor levels, similar levels of categorical variables were collapsed into broader categories.

Variables with a *p*-value below 0.10 in the association tests were included in conditional logistic regression models using the package survival [32]. To account for the lack of independence among samples from the same neighborhood, we included the neighborhood as a stratum.

### Sharing the evidence

In each neighborhood, a meeting was planned to share the results of the analysis of the water samples with neighbors and local leaders. These encounters took place at the Health Center of Barrio Chalet, at the Elementary Public School of La Vuelta del Paraguayo, and at the Popular Library of Colastiné Sur. Additionally, a meeting was scheduled to discuss the results with Ministry of Health agents.

### Ethics statement

All participants were informed about the objectives of the study and provided oral informed consent for their participation in the research activities; this consent was documented by the researchers during the encounters. Notes and records generated during the workshops were anonymized to remove personally identifiable information. All procedures complied with the ethical standards established by the Argentine Personal Data Protection Law (No. 25,326). The research project was reviewed and approved by the Ethics and Safety Advisory Committee for Research of the School of Biochemistry and Biological Sciences, Universidad Nacional del Litoral (Santa Fe, Argentina) (Act No. 03/17).

## Results

In all three neighborhoods the collective mapping encounters had good participation, with 6–10 neighbors attending each event. These encounters were also highly successful in the level of engagement in the activities proposed, the ease with which the trigger questions were addressed, and the consensus among participants on the location of key points on the map. This facilitated the straightforward identification of proper sampling sites.

The overlap of multiple icons served to identify hotspots in each of the three neighborhoods, leading to the collection of 11 samples per neighborhood. The sampling points represented a wide diversity of surface water features, including puddles or flooded areas in parks or streets (N = 13), sewers, storm drains, ditches and roadside channels (N = 11; Fig 2B), as well as lagoons, wetlands, rivers, or creeks (N = 9). Water samples exhibited a median pH of 6.9 (IQR: 6.7, 7.6), a median temperature of 14.8°C (IQR: 11.2, 15.7°C), a mean oxygen concentration of 5.5 mg/L (95% CI: 4.5-6.5 mg/L), a median conductivity of 10 µS/cm (IQR: 4, 159 µS/cm), a median specific conductivity of 13 µS/cm (IQR: 5, 203 µS/cm), and a median salinity of 0.00 ppt (IQR: 0.00, 0.10 ppt), with no significant differences observed among environmental sources (*p* > 0.05). However, samples from site CH exhibited significantly higher median temperatures (17.4°C, IQR: 15.3, 18.2°C) and pH (7.9, IQR: 6.9, 8.0) compared to sites LVP and CS (*p* < 0.001 and *p* = 0.005, respectively), while samples from site LVP had higher median salinity (0.1 ppt, IQR: 0.1, 0.1) than CH and CS (*p* = 0.005).

Most water bodies were in shade or semi-shade conditions (66.7%), with 33.3% exposed to direct sunlight. The majority of sites were surrounded by garbage accumulation (87.9%), with 55.2% containing low amounts of garbage and 44.8% classified as small dump yards. Debris or weeds were present in 24.2% of the sites, while stray dogs were observed in 81.3% and stray cats in 9.1%. Farm animals were detected in 36.4% of the sites, with horses and poultry present at 15.2% of sites each, cattle at 6.1%, and goats and rabbits at 3% each. No significant differences in the presence of garbage, debris, weeds, farm animals, or stray dogs or cats were detected among neighborhoods (*p* > 0.05).

**Fig 3:**
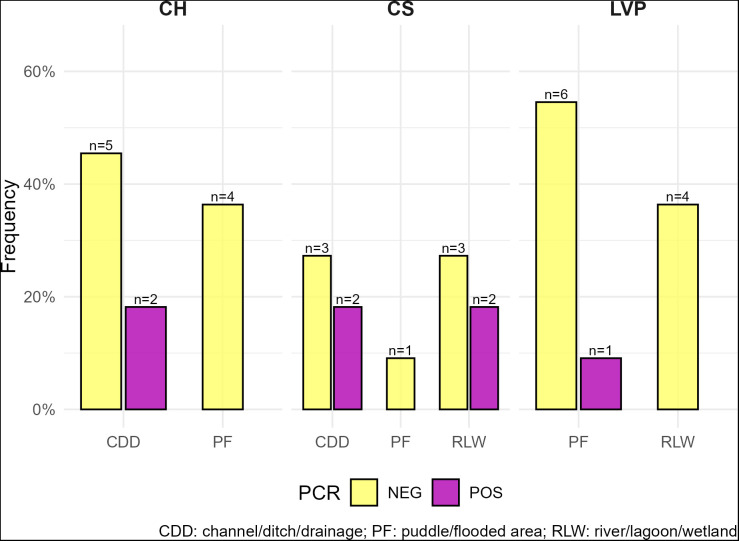
Frequency (%) of environmental samples positive for pathogenic *Leptospira* by sampling neighborhood and type of water body.

We successfully achieved the extraction of environmental DNA (eDNA) of good quality and the direct molecular detection of pathogenic *Leptospira* spp. in water samples from the three neighborhoods. The results of the qPCR indicated the presence of pathogenic *Leptospira* spp. in seven of the 33 samples analyzed (21.21%). The proportion of positive samples was 9.09% in LVP, 18.18% in CH and 36.36% in CS (Fig 3). For each positive sample, a fragment of 280 bp was sequenced and the BLAST algorithm implemented showed coincidences greater than 90% with the pathogenic species *Leptospira interrogans* for all amplicons obtained.

Analyses of the environmental conditions revealed no statistically significant associations between environmental variables and the presence of pathogenic leptospires. However, regarding physicochemical variables, a statistically significant association was observed between the presence of the bacterium and higher concentrations of dissolved Oxygen (*p* = 0.005), and marginally significant associations were observed with lower temperatures and the presence of stray dogs in the proximity of the water body (Table 2). These three variables were selected as explanatory variables for the conditional logistic regression models that also included neighborhood as a stratum. However, no inferences could be drawn from these models due to convergence problems.

**Table 2:** Frequency of samples positive to pathogenic *Leptospira* spp. by sampling neighborhood and environmental variables, Santa Fe, Argentina, 2019.

Characteristic	Overall (N = 33)^1^	NEG (N = 26)^1^	POS (N = 7)^1^	*p*-value^2^
Neighborhood				0.4
CH	11 (33.3%)	9 (34.6%)	2 (28.6%)	
CS	11 (33.3%)	7 (26.9%)	4 (57.1%)	
LVP	11 (33.3%)	10 (38.5%)	1 (14.3%)	
Type of water body				0.3
roadside channel/ditch/ drainage	12 (36.4%)	8 (30.8%)	4 (57.1%)	
puddle/flooded area	12 (36.4%)	11 (42.3%)	1 (14.3%)	
river/lagoon/wetland	9 (27.3%)	7 (26.9%)	2 (28.6%)	
pH				0.7
acid (<7)	18 (54.5%)	15 (57.7%)	3 (42.9%)	
basic (>7)	15 (45.5%)	11 (42.3%)	4 (57.1%)	
Temperature				0.085
10.4-14.8°C	17 (51.5%)	11 (42.3%)	6 (85.7%)	
14.8-19.9°C	16 (48.5%)	15 (57.7%)	1 (14.3%)	
Dissolved Oxygen (mg/l)				**0.005**
1.1 - 3.97	9 (27.3%)	7 (26.9%)	2 (28.6%)	
3.97 - 5.96	8 (24.2%)	8 (30.8%)	0 (0.0%)	
5.96 - 7.7	8 (24.2%)	8 (30.8%)	0 (0.0%)	
7.7 - 11.1	8 (24.2%)	3 (11.5%)	5 (71.4%)	
Compensated conductivity (µS/cm)				>0.9
1.3 - 4.8	9 (27.3%)	7 (26.9%)	2 (28.6%)	
4.8 - 13.3	8 (24.2%)	7 (26.9%)	1 (14.3%)	
13.3- 203	8 (24.2%)	6 (23.1%)	2 (28.6%)	
> 203	8 (24.2%)	6 (23.1%)	2 (28.6%)	
Conductivity (µS/cm)				0.8
1 - 3.7	9 (27.3%)	8 (30.8%)	1 (14.3%)	
3.7 - 9.8	8 (24.2%)	6 (23.1%)	2 (28.6%)	
9.8 - 159	8 (24.2%)	6 (23.1%)	2 (28.6%)	
>159	8 (24.2%)	6 (23.1%)	2 (28.6%)	
natural light				0.2
direct sunlight	11 (33.3%)	7 (26.9%)	4 (57.1%)	
shade/dappled shade	22 (66.7%)	19 (73.1%)	3 (42.9%)	
Salinity (ppt)				0.3
0	21 (63.6%)	17 (65.4%)	4 (57.1%)	
0.1	7 (21.2%)	6 (23.1%)	1 (14.3%)	
0.2	2 (6.1%)	1 (3.8%)	1 (14.3%)	
0.3	2 (6.1%)	2 (7.7%)	0 (0.0%)	
0.6	1 (3.0%)	0 (0.0%)	1 (14.3%)	
Presence of garbage	29 (87.9%)	23 (88.5%)	6 (85.7%)	>0.9
Amount of garbage				0.7
high	13 (44.8%)	11 (47.8%)	2 (33.3%)	
low	16 (55.2%)	12 (52.2%)	4 (66.7%)	
Presence of debris	8 (24.2%)	7 (26.9%)	1 (14.3%)	0.7
Presence of weeds	8 (24.2%)	6 (23.1%)	2 (28.6%)	>0.9
Presence of stray dogs	26 (81.3%)	22 (88.0%)	4 (57.1%)	0.10
Presence of poultry	5 (15.2%)	4 (15.4%)	1 (14.3%)	>0.9
Presence of livestock	8 (24.2%)	6 (23.1%)	2 (28.6%)	>0.9

^1^n (%); ^2^ Fisher’s exact test.

The results of the environmental analyses were later presented and discussed in meetings with community members and local health workers. These exchanges provided an opportunity to collectively review the findings, relate them to local experiences of flooding and environmental conditions, and reflect on possible actions to reduce exposure risks in the identified areas.

## Discussion and conclusions

### Molecular detection of pathogenic *Leptospira* spp. in urban water samples

This is the first report of pathogenic *Leptospira* in urban water bodies from the city of Santa Fe, Argentina, a city where leptospirosis is endemic [15]. We detected the DNA of the bacteria in 7 out of 33 samples. These samples came from a diverse array of water bodies, including flooded areas, sewers, storm drains, ditches, and lagoons. Our analysis identified dissolved oxygen as an environmental variable associated with the presence of the bacterium. This association should be interpreted with caution, as dissolved oxygen is highly temporally variable that is rarely evaluated in environmental studies of *Leptospira* [33,34] and has not consistently been associated with its occurrence (e.g., [35,36]). Given the limited number of samples and the non-probabilistic sampling strategy, these findings should be considered exploratory and cannot be used to estimate environmental prevalence or extrapolate beyond the sampled locations. Further studies with larger sample sizes would be useful to better characterize the environmental conditions that favor *Leptospira* persistence, particularly in stagnant or poorly circulating waters that are common in flood-prone areas of the region.

By using qPCR, a technique considered one of the most promising method for the detection of the bacterium in environmental samples [28,37–40], this study contributes to a better understanding of the occurrence of pathogenic *Leptospira* in urban water bodies. However, in Argentina most studies that look for the bacteria in environmental samples use isolation by culture, but this method detects mostly saprophytic species and, with less success, some pathogenic ones [41–45]. As a result, current knowledge of pathogenic leptospires in environmental samples in Argentina remains limited, and the findings presented here contribute to filling an important gap in understanding the ecology of the bacterium in our region.

The proportion of positive samples found here falls within the range reported in other studies conducted in South America, including 18.8% in urban water samples from Chile [46], and values exceeding 30% in studies from Brazil [28, 47]. It is important to note that in these studies, nucleic acid extraction technologies targeting microorganisms were employed. These approaches offer greater sensitivity, specificity, and time efficiency compared with traditional microbiological methods. However, the costs associated with these molecular technologies remain a significant barrier to their widespread adoption in resource-limited settings [48].

One of the advantages of the protocols used in our study is that they offer an affordable option for governmental laboratories in countries that may not have the resources to cover the high cost of commercial kits. These entities, such as the Administración Nacional de Laboratorios e Institutos de Salud (ANLIS) in Argentina, can benefit from the development and calibration of these protocols and implement them in efforts to monitor the prevalence of this bacterium in water bodies, in order to improve measures to prevent leptospirosis. The use of relatively simple molecular detection approaches may be particularly relevant in low- and middle-income countries, where access to specialized laboratory infrastructure and reagents can be limited. Conducting scientific research in Latin America is often shaped by structural constraints affecting funding and infrastructure [49,50], making cost-effective diagnostic and surveillance tools especially valuable for addressing neglected diseases such as leptospirosis.

### Leptospirosis through a participatory research approach

The environmental detection of *Leptospira* reported in this study is closely linked to the participatory approach used, as local residents played an active role in identifying sampling sites. Their collective knowledge allowed the identification of areas that were recognized as potential environmental risk hotspots for *Leptospira*. To our knowledge, few studies have applied participatory approaches to guide environmental sampling for the detection of *Leptospira* in natural water bodies, Community involvement in the research process is known to offer multiple advantages [51,52], and in our case, it proved particularly valuable for identifying locations where environmental conditions may favor exposure to leptospires. Residents’ detailed knowledge of the territory—including areas where water accumulates, sites with frequent animal activity, and places where people interact with environmental water— not only facilitated sample collection but also helped them make direct connections with the typical transmission pathway—exposure to contaminated water or soil [9].

In Santa Fe, leptospirosis outbreaks often follow flooding events caused by torrential rainfall or river overflow [15], with most epidemics historically coinciding with El Niño periods [22,53]. Our study took place shortly after the 2018–2019 El Niño episode, which produced significant rainfall and flooding in the region, although no active flooding was present during the sampling period. This El Niño event triggered a notable leptospirosis outbreak during the first three weeks of 2019 [54], with 48 cases confirmed in Santa Fe, representing a percentage increase of 71.4% compared to 2018 [55].

Zoonotic diseases disproportionately affect marginalized communities [56,57]. In Santa Fe, geographic vulnerability and social vulnerability interact and influence each other [25]. A recent study conducted by Cristaldi et al. [20] based on literature and expert knowledge found that the suitability for human leptospirosis in Santa Fe increased from downtown areas towards peri-urban and suburban areas. Consistent with this spatial pattern, Avalos et al. [58] reported that the incidence of leptospirosis in the city was positively associated with the Health Vulnerability Index, indicating a higher burden of disease in areas with greater socio-sanitary vulnerability. Poverty and marginalization in the neighborhoods that participated in this study create scenarios in which people are more frequently exposed to the bacteria and have fewer resources to prevent or mitigate the risks they face, especially those associated with severe impacts from floods [59]. Access to healthcare services in these neighborhoods is often limited, with some areas lacking permanent health facilities and residents relying on distant primary care centers. Previous studies in these neighborhoods indicate that information about leptospirosis—including its symptoms and transmission pathways—is not widely disseminated [60].

Diseases that are mainly prevalent among impoverished communities and that have significant impact on their health and socioeconomic conditions are considered by the World Health Organization as Neglected Tropical Discease (NTDs [61]). Surprisingly, leptospirosis is not on the list of the 20 NTD that the World Health Organization (WHO) is pursuing to prevent, control, eliminate or eradicate.

In Argentina, leptospirosis is considered an emerging public health problem (CCLA - AAVLD, 2002; MSAL, 2007, 2014) and is included among the notifiable diseases (National Law No. 15 465/1960). However, leptospirosis is under-reported due to misdiagnosis, especially during dengue epidemics, lack of awareness, and the difficulties in obtaining a second blood sample for a confirmatory diagnosis. For the aforementioned reasons, the information collected by the national health system on the population affected by leptospirosis is full of discrepancies and gaps [20]. According to Martins & Spink [13], these discrepancies and informational gaps generate an invisibility of those that are more at risk of contracting the disease, thus precluding that preventive measures and treatment reach the right target. Therefore, leptospirosis can be considered a doubly neglected disease.

The strategies to control zoonoses are usually organized vertically, i.e., they respond to priorities or programs developed by public officials and authorities, without articulation with members of the communities that are more familiar with the reality and living conditions of the people most vulnerable to these diseases [6]. This type of approach may result in zoonosis control strategies that are suboptimal or even inadequate for those who need them most, mostly because they involve measures that are impossible to be adopted by those at higher risk. Local people know what preventive measures are possible and realistic in their living context and with the available resources [62]. During the workshops conducted with the residents of the sectors where this study took place, participants frequently pointed out that several preventive measures promoted in governmental communication materials were difficult to implement in their daily activities. For instance, fishermen explained that recommendations such as avoiding contact with stagnant water or wearing high rubber boots were not feasible in their work context. They often search for small bait fish among aquatic vegetation in wetlands, which requires direct contact with water, and wearing boots while boating was considered dangerous because it could increase the risk of drowning. These observations illustrate how preventive recommendations designed without considering local livelihoods may overlook important practical constraints. This also underscores the importance of incorporating community perspectives in both disease control strategies and the research processes that inform them [5,63].

A similar top-down logic is also common in scientific research on zoonoses, where decisions about research design and implementation are often made primarily by researchers, with little involvement of the local communities who are directly affected by these diseases. In particular, in research related to the detection of *Leptospira* in environmental samples, the selection of soil and water sampling points rarely involves local residents, despite their valuable knowledge of the territory. The criteria that are typically used for selecting sampling points are the occurrence of leptospirosis cases, rat infestations, and poor waste management [64], researchers’ considerations of places where they assume abundant human and animal activity [65], or the implementation of a stratified sampling design that takes into account predefined distances [47]. In contrast, integrating local knowledge can help identify context-appropriate solutions and better understand their potential strengths and limitations [51]. Harnessing local knowledge through participatory methods can reveal risk factors that arise from social roles and cultural practices, and improve epidemiological models by providing information about local contexts [66].

Following a participatory approach, community members were actively involved in different stages of the research process. First, a series of workshops were held to establish an open knowledge dialogue. Similar workshops have been implemented in participatory research initiatives aimed at the control and prevention of neglected diseases such as dengue, leptospirosis and leishmaniasis [67–69]. During these workshops, participants shared knowledge and discussed neighborhood history, environmental conditions, and health concerns, fostering a dialogue between local experiences and scientific perspectives and preparing the ground for the subsequent identification of locations potentially associated with leptospirosis risk. Building on these discussions, a collaborative mapping exercise was conducted to identify potential hotspots for environmental surveillance. This participatory approach incorporates local knowledge and perspectives that may go unnoticed in traditional top-down research designs [68] and enables communities to analyze local problems and contribute to identify potential solutions [69]. Our study contributes to bridging participatory mapping approaches with environmental sampling for the molecular detection of *Leptospira* in urban water bodies, an integration that has rarely been documented.

Subsequent community meetings helped organize the sampling activities at the sites selected during the mapping exercise and allowed participants to coordinate tasks collectively. This collaborative organization fostered a sense of shared responsibility among participants and facilitated active involvement in both the planning and implementation of the research activities. Such participatory dynamics have been highlighted as important features of community-based research approaches that seek to integrate local knowledge with scientific investigation [4,51].

Our goal was to collectively evaluate the risk of leptospirosis by working with neighbors and local leaders to identify places where people could be exposed to the bacteria. Using this collaborative approach, we were able to detect the presence of the bacteria in flooded areas, sewers, storm drains, ditches, and lagoons. As described above, these findings were subsequently discussed with community members, creating a space for dialogue between scientific evidence and local knowledge. This process resembles what has been described as a “dialogue of evidences” in which different forms of knowledge are articulated to support the collective interpretation of environmental health risks [67,70]. The discussions during the workshops and meetings also stimulated local initiatives aimed at reducing exposure risks, including improvements in waste management in some areas and the establishment of a weekly health post in La Vuelta del Paraguayo. More broadly, our findings illustrate how community knowledge can contribute to environmental surveillance of zoonotic pathogens by helping identify locally relevant sites for investigation and fostering dialogue between researchers, public health institutions, and residents. Such approaches can complement conventional surveillance strategies by integrating local knowledge with scientific tools for pathogen detection. This participatory approach not only proved effective in identifying potential sources of leptospiral infections but also provided an opportunity to raise awareness about the disease and promote collective dialogue, including with decision makers, on strategies to reduce exposure risk. The results of this research are not only an important precedent for our region, but also a starting point for the application of monitoring and surveillance strategies in regions of Argentina with similar environmental and social characteristics.

## Supporting information

Striking image 1 Flooded area in the neighborhood of Colastiné Sur, Santa Fe, Argentina, during the 2018–2019 river overflow. After temporarily leaving their home due to rising water levels, this couple returned to check the condition of their belongings, crossing stagnant floodwaters despite the associated health risks. On the right, an electric fence contains horses relocated by local residents to the remaining dry areas along the embankment. The image illustrates how flooding, precarious infrastructure, and everyday livelihood practices converge to shape exposure to waterborne zoonotic diseases such as leptospirosis. Credit: Photo taken by MAP. This image can publish under the Creative Commons Attribution License (https://creativecommons.org/licenses/by/4.0).
(JPG)

Striking image 2 Community members and researchers collecting environmental water samples and measuring physicochemical variables in Barrio Chalet, Santa Fe, Argentina, as part of a participatory environmental surveillance initiative for leptospirosis. Through collaborative mapping and discussion workshops, residents identified this garbage-filled stagnant drainage channel along one of the neighborhood’s main streets as a potential exposure site. Molecular analyses later confirmed the presence of pathogenic *Leptospira* in the collected water sample, illustrating the value of integrating local knowledge with environmental pathogen detection. Credit: Photo taken by MAP. This image can be published under the Creative Commons Attribution License (https://creativecommons.org/licenses/by/4.0). The individuals pictured in this image have provided written informed consent (as outlined in PLOS consent form) to publish their image alongside the manuscript.
(JPG)
