# Supplementary figures and images for: Detection of pathogenic leptospires in water bodies in neighborhoods vulnerable to flooding, through a participatory research approach in Santa Fe (Argentina)
Source: PLOS Glob Public Health. 2026 Jun 8;6(6):e0006447. doi: 10.1371/journal.pgph.0006447 (PMC13245871; doi:10.1371/journal.pgph.0006447)

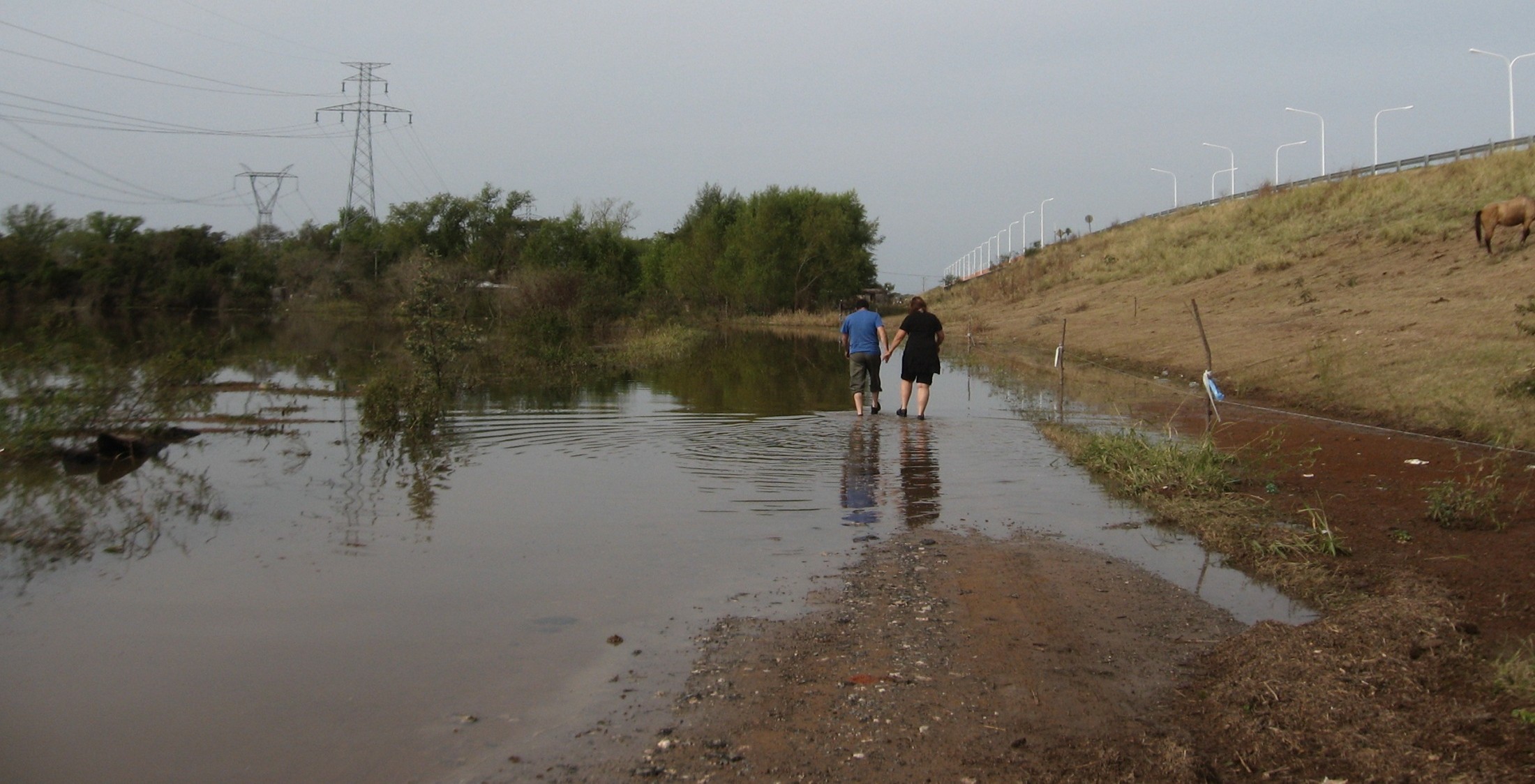

Supplement: Striking image 1 — After temporarily leaving their home due to rising water levels, this couple returned to check the condition of their belongings, crossing stagnant floodwaters despite the associated health risks. On the right, an electric fence contains horses relocated by local residents to the remaining dry areas along the embankment. The image illustrates how flooding, precarious infrastructure, and everyday livelihood practices converge to shape exposure to waterborne zoonotic diseases such as leptospirosis. Credit: Photo taken by MAP. This image can publish under the Creative Commons Attribution License (https://creativecommons.org/licenses/by/4.0). (JPG) [file pgph.0006447.s001.jpg]

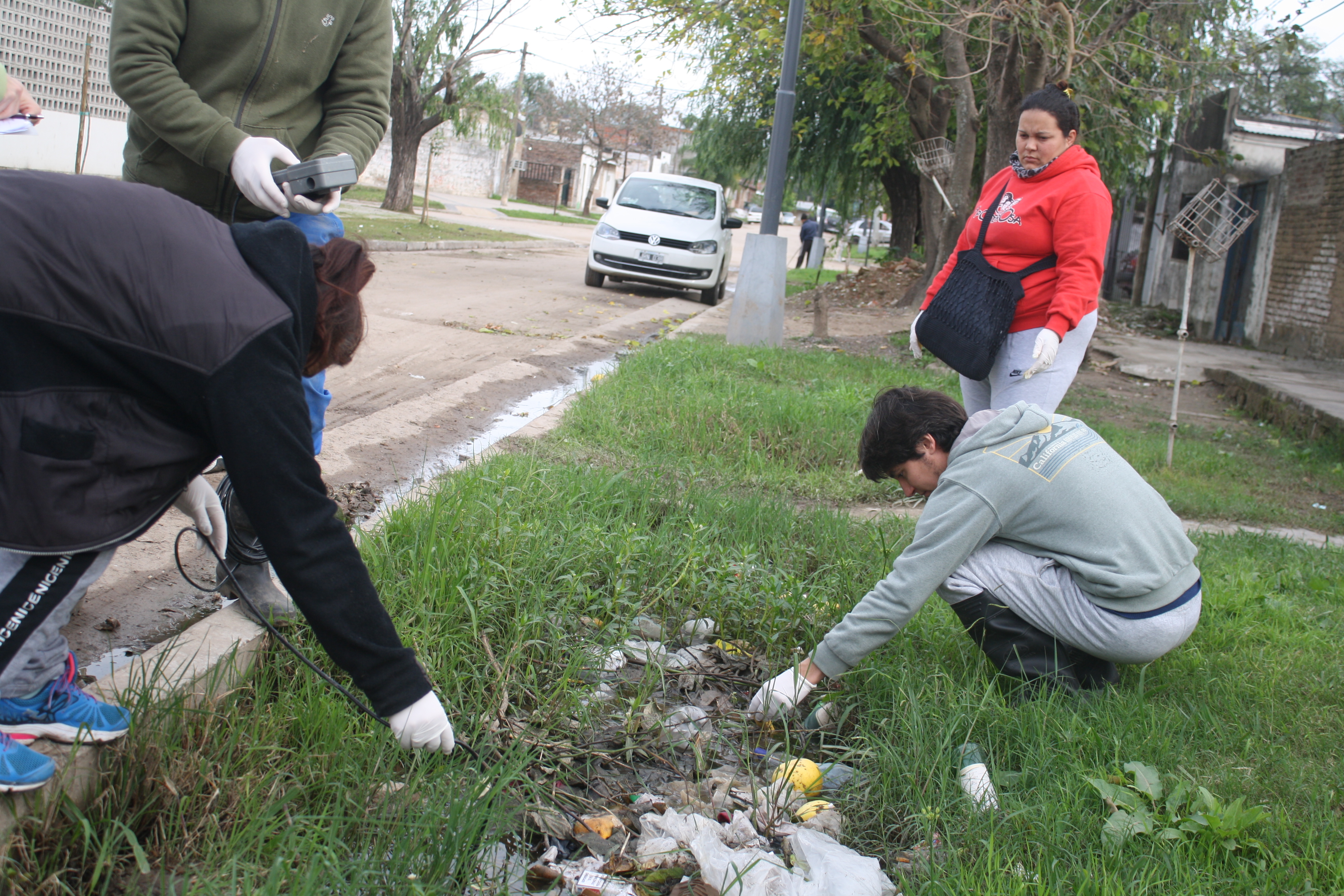

Supplement: Striking image 2 — Through collaborative mapping and discussion workshops, residents identified this garbage-filled stagnant drainage channel along one of the neighborhood’s main streets as a potential exposure site. Molecular analyses later confirmed the presence of pathogenic Leptospira in the collected water sample, illustrating the value of integrating local knowledge with environmental pathogen detection. Credit: Photo taken by MAP. This image can be published under the Creative Commons Attribution License (https://creativecommons.org/licenses/by/4.0). The individuals pictured in this image have provided written informed consent (as outlined in PLOS consent form) to publish their image alongside the manuscript. (JPG) [file pgph.0006447.s002.jpg]
